# The role of Notch ligand Jagged1 in osteosarcoma proliferation, metastasis, and recurrence

**DOI:** 10.1186/s13018-021-02372-y

**Published:** 2021-03-29

**Authors:** Jianping Zhang, Na Li, Siyu Lu, Yanling Chen, Lequn Shan, Xingcheng Zhao, Yongqing Xu

**Affiliations:** 1 Department of Orthopedic Surgery, 920th Hospital of Joint Logistics Support Force, Daguan Road 212#, Kunming, 650032 China; 2 Department of Oncology, Tangdu Hospital, Fourth Military Medical University, Xi’an, 710032 China; 3 Department of Orthopedic Surgery, Tangdu Hospital, Fourth Military Medical University, Xi’an, 710032 China; 4 School of Aerospace Medicine, Fourth Military Medical University, Changle West Road 169#, Xi’an, 710032 China

**Keywords:** Osteosarcoma, Jagged1, Notch, Metastasis, Recurrence

## Abstract

**Background:**

Osteosarcoma is the most common primary bone cancer occurring in young adults and the 5-year survival rate of patients with metastatic osteosarcoma is less than 30% due to high metastatic recurrence and drug resistance. Notch is a highly conserved cell to cell signaling pathway in evolution, and Jagged1 is an important ligand of Notch. Although some studies have found that Notch receptors and ligands including Jagged1 were highly expressed in osteosarcoma tissues and osteosarcoma cells, the role of Jagged1 in osteosarcoma progression and metastasis are still not clear.

**Methods:**

Tumor tissues were collected from 68 patients and immunohistochemical staining was employed to group these patients by expression of Jagged1. Real-time quantitative PCR and Western blotting were used to detect the expression of Jagged1. We used siRNA to knockdown the expression of Jagged1 in F5M2 cells. Colony formation assay and MTT were employed to detect and analyze the proliferation of F5M2 cells with or without knockdown of Jagged1. Transwell assay were used to detect the migration and invasion of F5M2 cells.

**Results:**

In this study, we found that the high expression of Jagged1 is closely related to the metastasis and recurrence of osteosarcoma in 68 clinical specimens. The expression of Jagged1 in F5M2 cells with high metastasis was significantly higher than that in F4 cells with low metastasis. Knockdown of Jagged1 led to lower ability of proliferation, migration, and invasion in F5M2 cells.

**Conclusion:**

The high expression of Jagged1 is closely related to the metastasis and recurrence of osteosarcoma. Knockdown of Jagged1 significantly reduced the proliferation, migration, and invasion of osteosarcoma cells. Our results suggested that knockdown of Jagged1 may be a potentially effective treatment for metastatic osteosarcoma.

## Introduction

Osteosarcoma is the most common primary bone cancer occurring in young adults and the third highest cause of cancer-related death after leukemia and nervous-system cancers in children and adolescents under 20 years of age. Consistent with its high incidence in adolescents, osteosarcoma preferentially develops in the metaphyseal region of the most rapidly growing bones such as the distal femur, with approximately 60% of cases originating around knee area [1, 2]. Osteosarcoma is often highly aggressive with 20% patients presenting overt distal organ metastases (most are lung) at initial diagnosis [3, 4]. Over the past third decades, the 5-year survival for patients with localized disease has dramatically increased from less than 20% to 70% due to the combination of using surgery, neoadjuvant, and adjuvant chemotherapy. Unfortunately, for patients with metastatic or recurrent disease remain to have a poor prognosis, with only 30% surviving at 5 years. Respiratory failure resulted from pulmonary metastasis is the main cause of death in patients with osteosarcoma [5–7]. At present, the mechanisms underlying osteosarcoma metastasis is still limited. Along with extensive research in osteosarcoma, several biomarkers have been identified in osteosarcoma, such as aberrant modification of tumor suppressor-gene Rb [8], P53 genes [9], and oncogene c-MYC [10], c-FOS genes [11].

Notch signaling pathway is a highly conserved cell signal transduction system in evolution. By regulating cell proliferation, apoptosis, and differentiation, Notch signaling pathway plays an important role in a series of physiological and pathological processes, such as embryonic development, adult homeostasis maintenance, immune regulation, and disease generation. Notch signaling pathway is composed of three important functional structures, including Notch receptor, ligand, and nuclear transcription factor. There are four Notch receptors (Notch1, Notch2, Notch3, and Notch4) and five Notch ligands (Delta-like 1 (Dll1), Dll3, Dll4, Jagged1, and Jagged2 in mammals) [12, 13]. Notch signaling pathway plays a dual role in promoting and inhibiting tumorigenesis. Tumor metastasis is the main cause of recurrence and poor prognosis. Some studies have shown that the blocking of Notch signal destroys tumor vascular structure and promotes tumor metastasis [14–16]. Engin and Tanaka [17, 18] have found that many kinds of Notch signaling pathway molecules are highly expressed in osteosarcoma tissues and osteosarcoma cells. Blocking Notch signaling pathway with GSI (γ-secretase inhibitor) can inhibit the proliferation of osteosarcoma cells in vitro and tumorigenesis in vivo [19], which indicates that Notch signaling pathway is activated in osteosarcoma and plays a role of oncogene.

In this study, to investigate the relationship between Jagged1 and osteosarcoma metastasis and recurrence, a randomized and double-blind retrospective study was conducted in 68 patients. In addition, Jagged1 was interfered with siRNA transfection in a high metastatic osteosarcoma cell line to evaluate the effect of downregulation of Jagged1 on its proliferation, migration, and invasion. This study will lay a foundation for further understanding the mechanism of osteosarcoma development and provide a possible target for its treatment.

## Materials and methods

### Tissue specimens and patients

Tumor tissues were collected from 68 patients with osteosarcoma who received successful tumor resection or biopsy at the Department of Orthopedic Surgery of Tangdu Hospital, Fourth Military Medical University, from January 2007 to January 2009. There were 43 male and 25 female patients. Inclusion criteria are as follows: (1) who were confirmed osteosarcoma postoperative pathology; (2) who were performed osteosarcoma-resection operation firstly at the Department of Orthopedics, Tangdu Hospital, Fourth Military Medical University; (3) who were followed up for 5 years or relapsed or died;(4) who were volunteer for this study. Exclusion criteria are as follows: (1) who had dysfunction in vital organs or severe heart diseases; (2) who were performed operation after failure of chemotherapy or radiotherapy; and (3) who refused to sign consent information and provide their clinical data. The expression of Jagged1 in osteosarcoma tissues were examined by immunohistochemical stain and evaluated by two pathologists. The data was analyzed with clinical characteristic of patients. This study was approved by the Institutional Review Board of Tangdu Hospital, Fourth Military Medical University. Written informed consent was obtained from each patient or their legally authorized representative.

### Immunohistochemical staining

Immunohistochemical staining was performed with the Envision peroxidase complex method. Tissue sections (4 μm thick) were mounted on slides and then deparaffinized and rehydrated through xylene baths and graded concentrations of alcohol. After the paraffin sections being soaked in 0.3% hydrogen peroxide water, they were placed in 0.01 mol/L citrate buffer (pH 6.0) and boiled (95 °C, 15-20 min) for antigen retrieval. After the sections being blocked by BSA (bovine serum albumin), they were incubated by the primary antibody (Rabbit anti human Jagged1, Abcam, Cambridge, MA; Mouse anti β-actin, Sigma, St. Louis, MO) overnight and then the secondary antibody (horseradish peroxidase conjugated goat anti-rabbit IgG, Proteintech, Chicago, IL; horseradish peroxidase conjugated goat anti-mouse IgG, Proteintech, Chicago, IL) for 30 min. DAB (diaminobenzidine) chromogenic solution developed hematoxylin stains the nucleus.

### Real-time quantitative PCR

Cells were lysed with Trizol, and RNA (ribonucleic acid) was extracted with chloroform and isopropanol. The RNA was reverse-transcript into cDNA (complimentary deoxyribonucleic acid) after the concentration being determined. Real-time quantitative PCR (polymerase chain reaction) is completed with ABI 7500 instruments. The primers used were as follows: Jagged forward, 5′-GAGCTATTTGCCGACAAGGC-3′; Jagged reverse, 5′-GGAGTTTGCAAGACCCATGC-3′; β-actin forward, 5′-AGTTGCGTTACACCCTTTCTTG-3′; β-actin reverse, 5′-TCACCTTCACCGTTCCAGTTT-3′.

### Western blotting

The cells were lysed with RIPA (Beyotime, Shanghai, China) and the protein concentration was determined. Then sample buffer was added and the sample was boiled for 5 min. The protein sample was transferred to PVDF membrane after electrophoresis. Then the membrane was sealed with BSA and incubated with primary antibody overnight at 4 °C. Then the membrane was developed after incubation with secondary antibody. The primary antibody and secondary antibody were as before.

### MTT

Cells were digested with trypsin (BioCytoSci, Xi’an, China) and diluted to 1×10^4^ cells/ml. Two hundred microliters diluted cell suspension was added into 96-well plate, and 5 multiple pores were added to each experimental group. When the detection time point was reached, 20 μl MTT [3-(4,5-dimethylthiazol-2-yl)-2,5-diphenyltetrazolium bromide, Sigma, St. Louis, MO] solution (5 mg/ml) was added into each well, and then it was mixed and incubated in 37 °C carbon dioxide incubator for 4 h. After absorbing the culture medium, 150 ml DMSO (dimethyl sulfoxide, Sigma, St. Louis, MO) was added into each well. After shaking for 10 min, a 490-nm wavelength was selected for detection.

### Clone formation assay

Cells were digested with trypsin and diluted to 1×10^3^ cells/ml. Five hundred milliliters of the above cell suspension was added into 6-well plate, 2 ml RPMI (Roswell Park Memorial Institute) 1640 medium (Gibco, Waltham, MA) was added, and incubated in carbon dioxide incubator at 37 °C for 12 days. The culture medium was changed every 3 days and the colony formation was observed. When reaching the observation point, cells were fixed with alcohol for 20 min, dyed with crystal violet for 15 min, and finally photos were taken under the microscope.

### Cell migration assay

Fifty microliters/well serum-free medium containing 0.1% BSA was added into 24 well Transwell chamber (BD, San Jose, CA) and incubated in 37 °C carbon dioxide incubator for 30 min. Cells were cultured in serum-free RPMI 1640 for 24 h, digested by trypsin, and the cell concentration was adjusted to 1 × 10^5^ cells/ml. One hundred microliters of cell suspension was added to the upper layer of Transwell chamber, and 600 μl RPMI 1640 medium (Gibco, Waltham, MA) containing 20% fetal bovine serum (Sijiqing, Hangzhou, China) was added to the lower layer of Transwell chamber. The plates were incubated in 37 °C carbon dioxide incubator for 18 h. Transwell chamber was taken out, upper layer cells were wiped off, and the lower layer cells were fixed with alcohol and stained with crystal violet. Finally, they were observed and photographed under an inverted microscope.

### Cell invasion assay

Twenty-five microliters of Matrigel (diluted with RPMI 1640 in the ratio of 1:3) was added to each Transwell chamber. Fifty microliters/well serum-free medium containing 0.1% BSA was added into 24 well Transwell chamber (BD) and incubated in 37 °C carbon dioxide incubator for 30 min. Cells were cultured in serum-free RPMI 1640 for 24 h, digested by trypsin, and the cell concentration was adjusted to 3 × 10^5^ cells/ml. One hundred microliters of cell suspension was added to the upper layer of Transwell chamber, and 600 μl RPMI 1640 medium containing 20% fetal bovine serum was added to the lower layer of Transwell chamber. The plates were incubated in 37 °C carbon dioxide incubator for 18 h. Transwell chamber was taken out, upper layer cells were wiped off, and the lower layer cells were fixed with alcohol and stained with crystal violet. Finally, they were observed and photographed under an inverted microscope.

### Statistical analysis

Results are presented as the mean 6 SE (standard error). Significance was determined using the two-tailed paired Student *t* test. *P* < 0.05 was considered significant.

## Results

### The high expression of Jagged1 has a significant correlation with the metastasis and recurrence of osteosarcoma

**Fig. 1:**
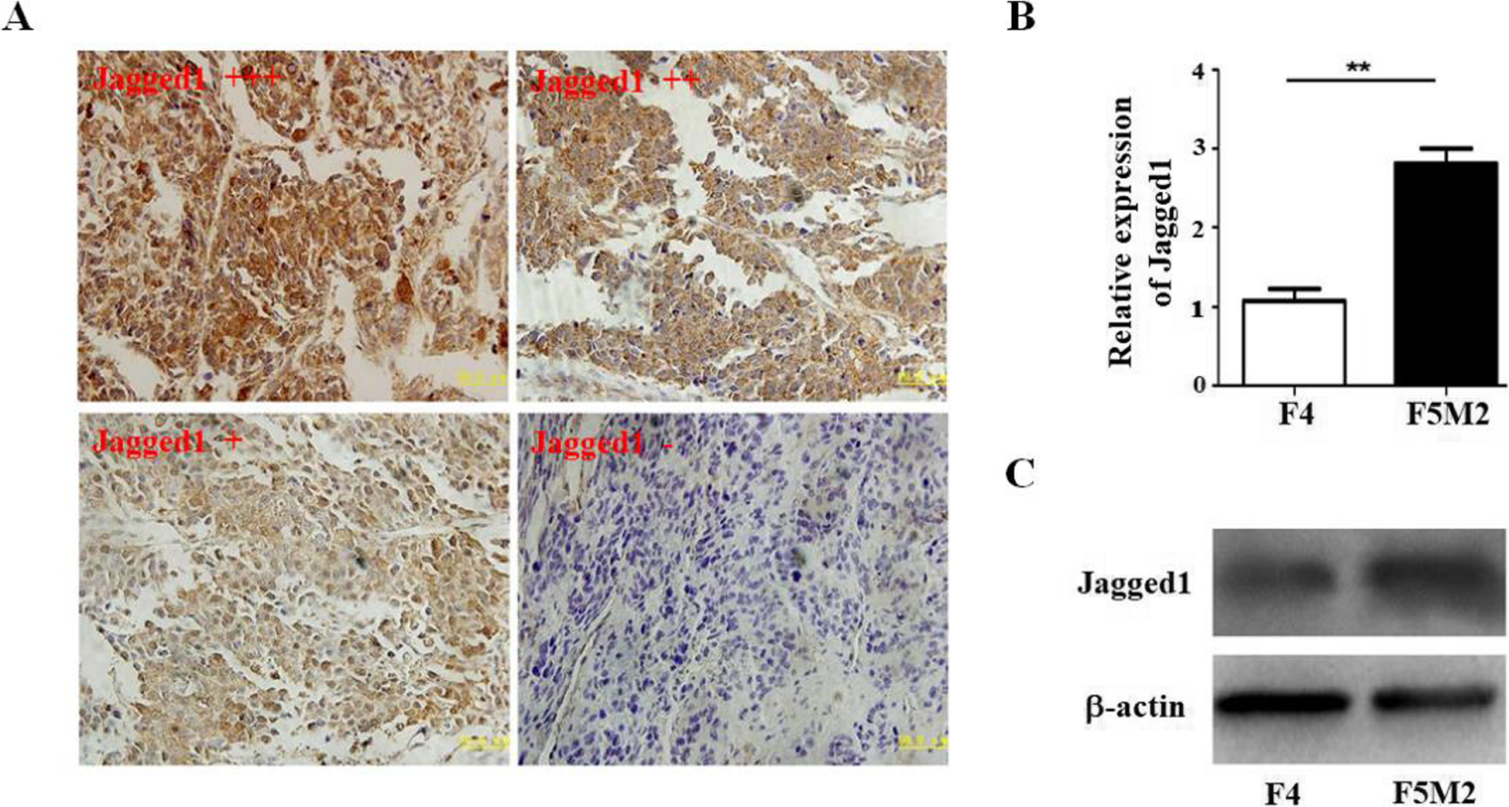
The expression of Jagged1 in and osteosarcoma specimens and cells. **a** Representative pictures of strong positive (+++), moderate positive (++), weak positive (+), and negative (−) staining of Jagged1 immunohistochemistry in osteosarcoma specimens. Scale bar = 50 μm. **b** Relative expression of Jagged1 mRNA in F4 osteosarcoma cells with low metastasis and F5M2 osteosarcoma cells with high metastasis detected by real-time quotative RT-PCR. **P<0.01. **c** The expression of Jagged1 protein in F4 osteosarcoma cells with low metastasis and F5M2 osteosarcoma cells with high metastasis detected by Western blotting

In order to explore the correlation between the expression of Jagged1 and the prognosis of osteosarcoma, we used immunohistochemical staining to detect 68 specimens. We divide the expression of Jagged1 into four categories: strong positive (+++), moderate positive (++), weak positive (+), and negative (−) (Fig. 1a). Immunohistochemical results indicated that Jagged1 expression was strongly positive in 69% osteosarcoma samples (47/68), it was more noteworthy that in 24 metastatic samples, the positive stain is 87.5% including 17 strong positive cases (+++), 4 moderate positive cases (++), whereas among non-metastatic samples, the positive stain is 59% (26/44), including 6 strong positive cases (+++) and 20 moderate positive cases (++). The results indicate that Jagged1 expression was positively correlated with tumor metastasis (*P*=0.0154) (Fig. 1a and Table 1).

**Table 1:**
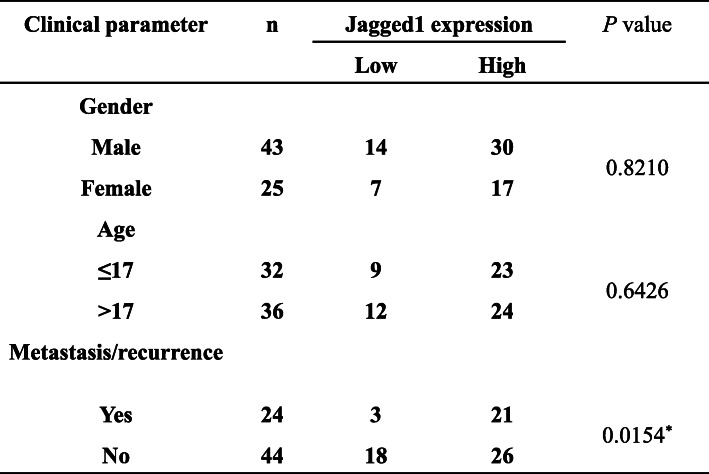
Relationships between the expression of Jagged1 and metastasis/recurrence in osteosarcoma patients

In our previous study, we established an osteosarcoma cell line SOSP-9607 (established ourselves, reference [20]) with high tumorigenicity rate by primary human osteosarcoma cells [20]. After 12 monoclonal cells were isolated by multiple dilution method, two cell lines with high metastasis (100% metastasis) and low metastasis (less than 10%) were selected, named F5M2 (established ourselves, reference [21]) and F4 (established ourselves, reference [21]) respectively. The results showed that the morphology of F5M2 and F4 cells was similar, but the proliferation and metastasis ability of F5M2 in vitro and in vivo was significantly higher than that of F4 [21]. We also detected the expression of Jagged1 in the osteosarcoma cell lines F4 (low metastasis beads) and F5M2 (high metastasis beads). Real-time PCR illustrated that Jagged1 expression is 3.8 times higher in F5M2 cells compare to F4 cells (Fig. 1b). Western blot results showed that Jagged1 expression level was higher in F5M2 cells than its expression in F4 cells (Fig. 1c).

### Knockdown of Jagged1 inhibits the proliferation of osteosarcoma cells

**Fig. 2:**
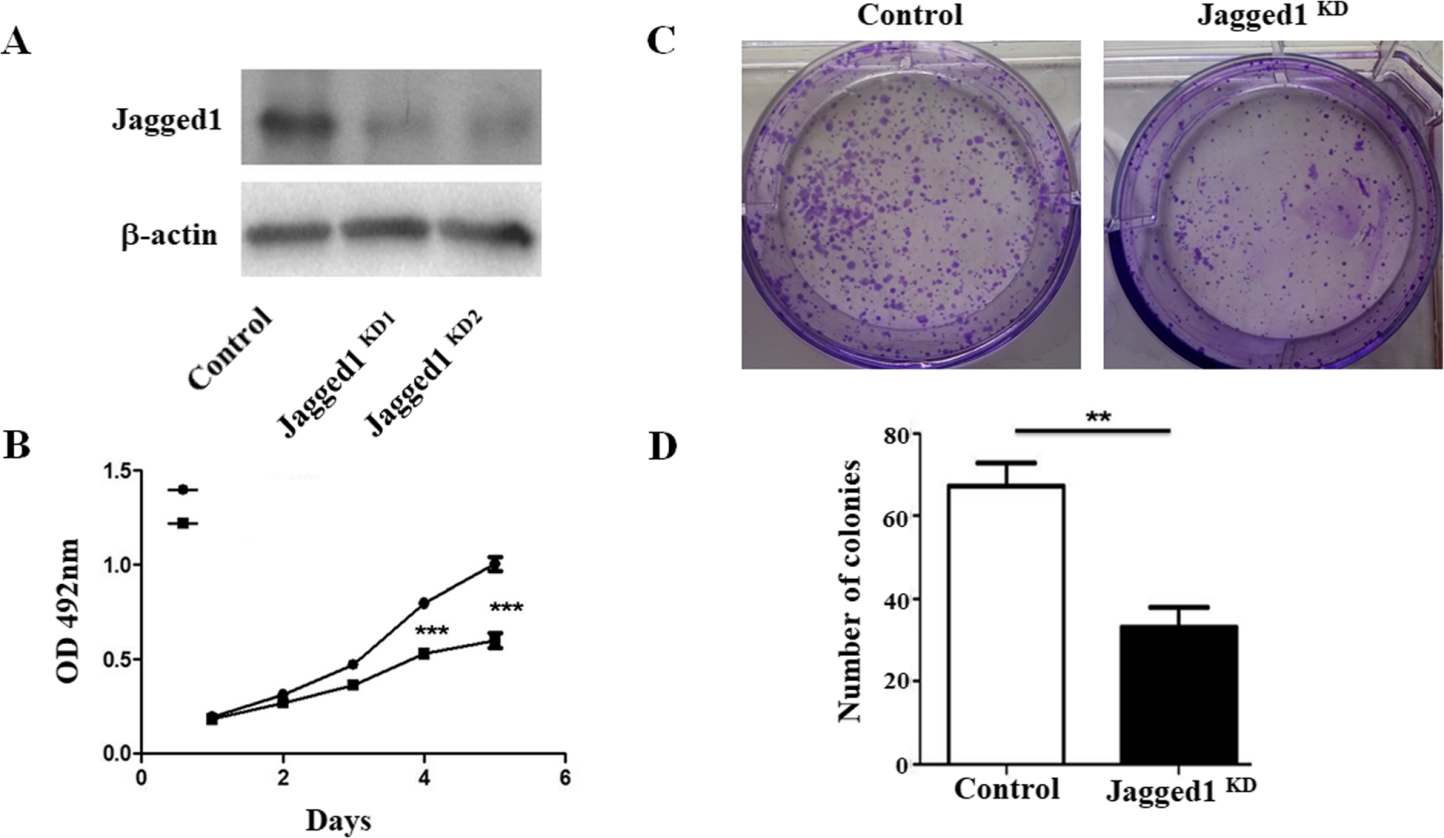
Knockdown of Jagged1 inhibits the proliferation of osteosarcoma cells. **a** Western blotting analysis of Jagged1 expression after transfecting Jagged1-siRNA or control into F5M2 cells. **b** MTT analysis of the proliferation of F5M2 after transfecting Jagged1-siRNA or control. **c** Cell colony forming analysis of the proliferation of F5M2 after transfecting Jagged1-siRNA or control. **d** The statistic of colony number of F5M2 after transfecting Jagged1-siRNA or control. **P<0.01

In order to test whether knockdown of Jagged1 affects the proliferation of osteosarcoma cells, we synthesized small interfering RNA of Jagged1. The results of Western blotting showed that siRNA2 has a better effect among small interfering RNAs in two siRNAs (small interfering RNAs) (Fig. 2a), so we will use siRNA2 in subsequent experiments. After transfecting Jagged1-siRNA into F5M2 cells, the expression of Jagged1 was effectively silenced by 80% compared to control-siRNA transfected cells (Fig. 2a). Silence Jagged1 expression significantly suppressed F5M2 cells proliferation as determined by MTT assay (Fig. 2b), and the number of cell colonies in Jagged1-siRNA group was extremely less than control group (67±6 vs 33±5) (Fig. 2c and d), the difference was significant. These results suggested that knockdown of Jagged1 inhibited the proliferation of osteosarcoma cells.

### Knockdown of Jagged1 inhibits the migration and invasion of osteosarcoma cells

**Fig. 3:**
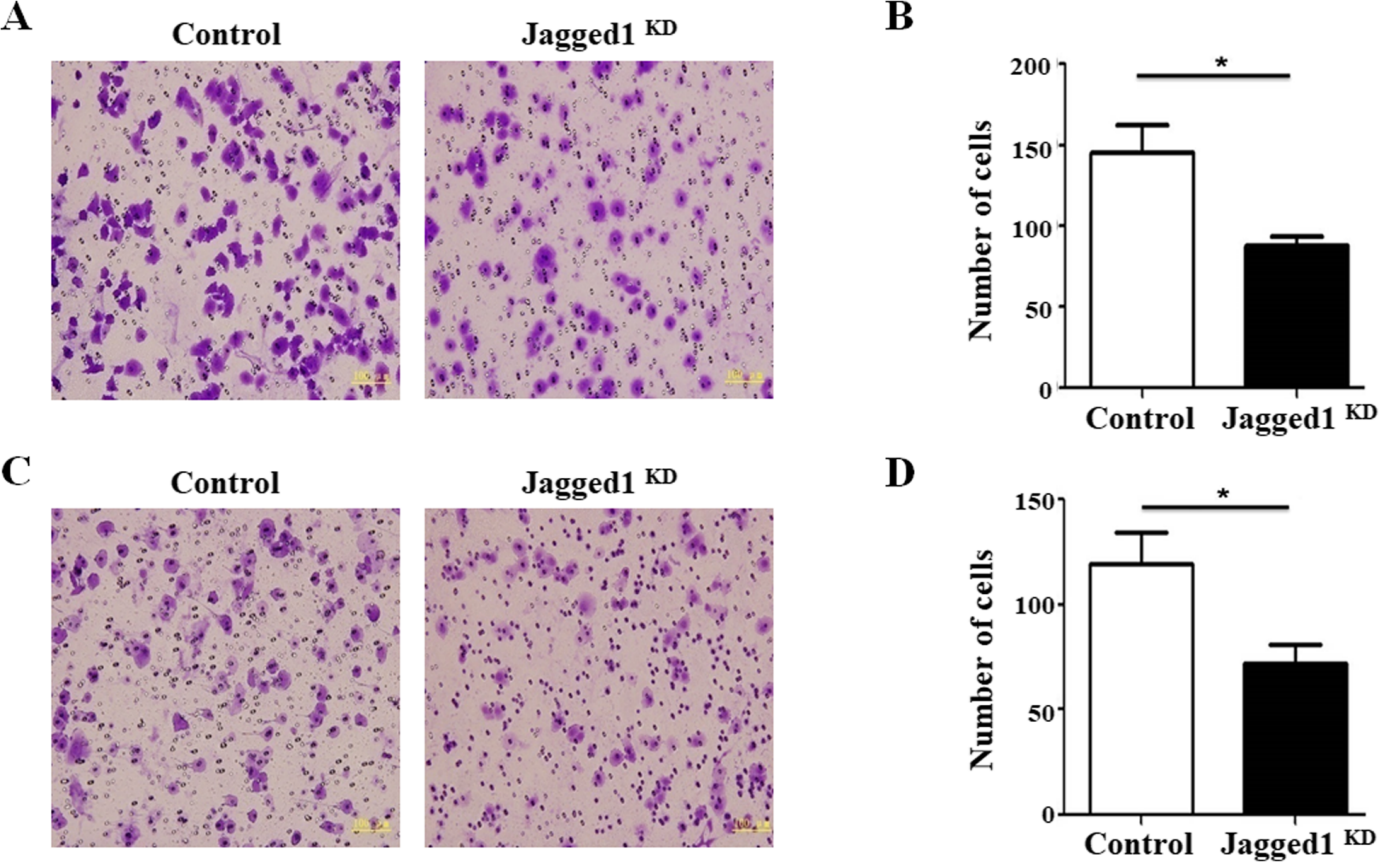
Knockdown of Jagged1 inhibits the migration and invasion of osteosarcoma cells. **a** The representative images of migrated F5M2 cells after transfecting Jagged1-siRNA or control and compared in (**b**). *P<0.05. **c** The representative images of invasive F5M2 cells after transfecting Jagged1-siRNA or control and compared in (**d**). *P<0.05

In order to test whether the knockdown of Jagged1 affects the metastasis and invasion of osteosarcoma cells, we used the Transwell to detect. Through preliminary experiments, we found that after 18 h of culture, some F5M2 cells could pass through the microporous membrane and adhere to the membrane. Therefore, we wiped off the non-migrating cells on the microporous membrane after 18 h of culture, and counted the number of migrated cells under a microscope after fixing and staining. The results showed that there were about (145±17) cells in each field on average in Jagged1 knockdown group, while the control group had (87±6) cells in each field on average (Fig. 3a and b).

The key step for tumor cell invasion and metastasis is to degrade the extracellular matrix and vascular basement membrane to open a pathway for tumors and tumors. Therefore, we added ECM (extracellular matrix) to the microporous membrane of Transwell chamber to simulate the tumor extracellular matrix environment. We observed the effect of silencing the Jagged1 gene on the penetration of tumor cells through the microporous membrane by degrading ECM glue. Through preliminary experiments, we found that tumor cells seeded in the upper chamber of Transwell could pass through the microporous membrane after 30 h of culture. We wiped off the upper cells on the microporous membrane after 30 h of culture, and counted the number of migrated cells under a microscope after fixing and staining. The results showed that there were about 119±5 cells in each field on average in Jagged1 knockdown group, while the control group had 72±5 cells in each field on average (Fig. 3c and d).

These results suggested that knockdown of Jagged1 inhibited the migration and invasion of osteosarcoma cells.

## Discussion

Many studies have shown that Notch signaling plays an important role in the occurrence and development of osteosarcoma. Qin et al. reported that inhibition Notch signaling with GSI effectively inhibits osteosarcoma proliferation and metastasis in vivo and in vitro by inhibiting Erk phosphorylation [19]. Gao et al. reported that Notch-1 overexpression increased the viability of OS cells through upregulation of Cdc20 expression [22]. Of course, there are some reports to the contrary. Ren et. al. reported that the Notch signal transduction pathway participates in tumor occurrence and growth with a negative role by maintaining Th1/Th2 balance [23]. However, the role of Notch ligand Jagged1 in the tumorigenesis of osteosarcoma is still unclear. Both Engin and Tanaka have found that Notch ligand Jagged1 is highly expressed in osteosarcoma [17, 18]. These studies suggest that Jagged1 may play an important role in the metastasis and recurrence of osteosarcoma. Therefore, we have carried out in-depth research on this issue.

Notch signaling pathway is involved in the regulation of cell cycle in a variety of tumor cells. Inhibition of Notch signaling pathway in T-ALL (T cell acute lymphoblastic leukemia) can block tumor cells in G1 phase. Further studies have found that Cyclin D3, CDK4, and CDK6 are direct target genes of Notch [24]. In pancreatic cancer, Notch1 inhibits the expression of p21 and p27 by activating Cyclin D1 expression, and promotes tumor cell cycle [25]. Jagged1 also plays an important role in tumor cell cycle regulation. Recent studies have shown that Cyclin D1 is the direct target gene of Jagged1 dependent Notch signaling pathway activation in breast cancer cells, and knockdown of Jagged1 can inhibit cell proliferation by delaying the transition from G1 to S phase [26]. Dai et al. also found that inhibition of Jagged1 could effectively reduce the expression of cyclin D1, cyclin E, and c-myc in colorectal cancer cell, and block tumor cells in G1 phase [27]. Some studies have also suggested that in prostate cancer cells, inhibition of Jagged1 gene expression causes tumor cells to block in S phase [28]. These different results may be due to the cell-dependent expression of Notch signaling pathway. However, in general, these studies have confirmed that Notch signaling pathway, including Jagged1, plays an important role in the regulation of tumor cell cycle. Our study in osteosarcoma cells also found that inhibition of Jagged1 expression in tumor cells can significantly reduce the proliferation activity of F5M2 cells and inhibit its division ability. However, the widely recognized Cyclin D1 gene is not regulated by Jagged1 in our research system. Therefore, due to the complexity of Notch signaling pathway, the molecular mechanism of Jagged1 knockdown on inhibiting the proliferation of osteosarcoma cells needs to be further explored.

Clinical studies found that about 20% of osteosarcoma patients also found metastasis at the first visit, and about 30% of patients with in situ tumor died of tumor metastasis within 5 years. In recent years, in-depth study of the molecular mechanism of osteosarcoma metastasis and exploring effective molecular targets for prevention and treatment of osteosarcoma metastasis have become the focus of basic and clinical research of osteosarcoma in recent years. In this study, we used F5M2 cells with high metastatic characteristics, and verified the effect of Notch signaling pathway on the invasion and metastasis of osteosarcoma cells in vitro. We found that knockdown of Jagged1 gene, which was highly expressed in F5M2 cells, significantly inhibited the migration and invasion of tumor cells. Although we failed to carry out animal model experiment of osteosarcoma metastasis and Jagged1 downstream target molecular screening experiment, our experimental results still provide reliable experimental data for exploring the relationship between Notch signaling pathway and osteosarcoma, and provide a solid foundation for further experimental research.

## Data Availability

The datasets used during the current study are available from the corresponding author on reasonable request.

## Abbreviations

BSA: Bovine serum albumin
cDNA: Complimentary deoxyribonucleic acid
DAB: Diaminobenzidine
Dll: Delta-like
DMSO: Dimethyl sulfoxide
ECM: Extracellular matrix
GSI: γ-Secretase inhibitor
MTT: 3-(4,5-dimethylthiazol-2-yl)-2,5-diphenyltetrazolium bromide
PCR: Polymerase chain reaction
RNA: Ribonucleic acid
RPMI: Roswell Park Memorial Institute
SE: Standard error
siRNA: Small interfering RNA
T-ALL: T cell acute lymphoblastic leukemia
